# Deciphering Metabolic Currencies That Support Marine Microbial Networks

**DOI:** 10.1128/mSystems.00763-21

**Published:** 2021-08-24

**Authors:** Bryndan P. Durham

**Affiliations:** a Department of Biology, Genetics Institute, University of Florida, Gainesville, Florida, USA

**Keywords:** cell signaling, cell-cell interaction, marine microbiology, metabolic regulation, metabolomics, microbial networks, phytoplankton, sulfonates, sulfur metabolism

## Abstract

Microbes are omnipresent in the biosphere and perform biological and chemical processes critical to ecosystem function, nutrient cycling, and global climate regulation. In the ocean, microbes constitute more than two-thirds of biomass with abundances reaching over one million microbial cells per milliliter of seawater. Our understanding of the marine microbial world has rapidly expanded with use of innovative molecular and chemical ‘omics tools to uncover previously hidden taxonomic diversity, spatiotemporal distributions, and novel metabolic functions. Recognition that specific microbial taxa cooccur in consistent patterns in the ocean has implicated microbe-microbe interactions as important, but poorly constrained, regulators of microbial activity. Here, I examine cooperative interactions among marine plankton, with a focus on the metabolic “currencies” that establish microbial partnerships in the surface-ocean trade economy. I discuss current and future directions to study microbial metabolic interactions in order to strengthen our understanding of ecosystem interdependencies and their impact on ocean biogeochemistry.

## COMMENTARY

**FIG 1:**
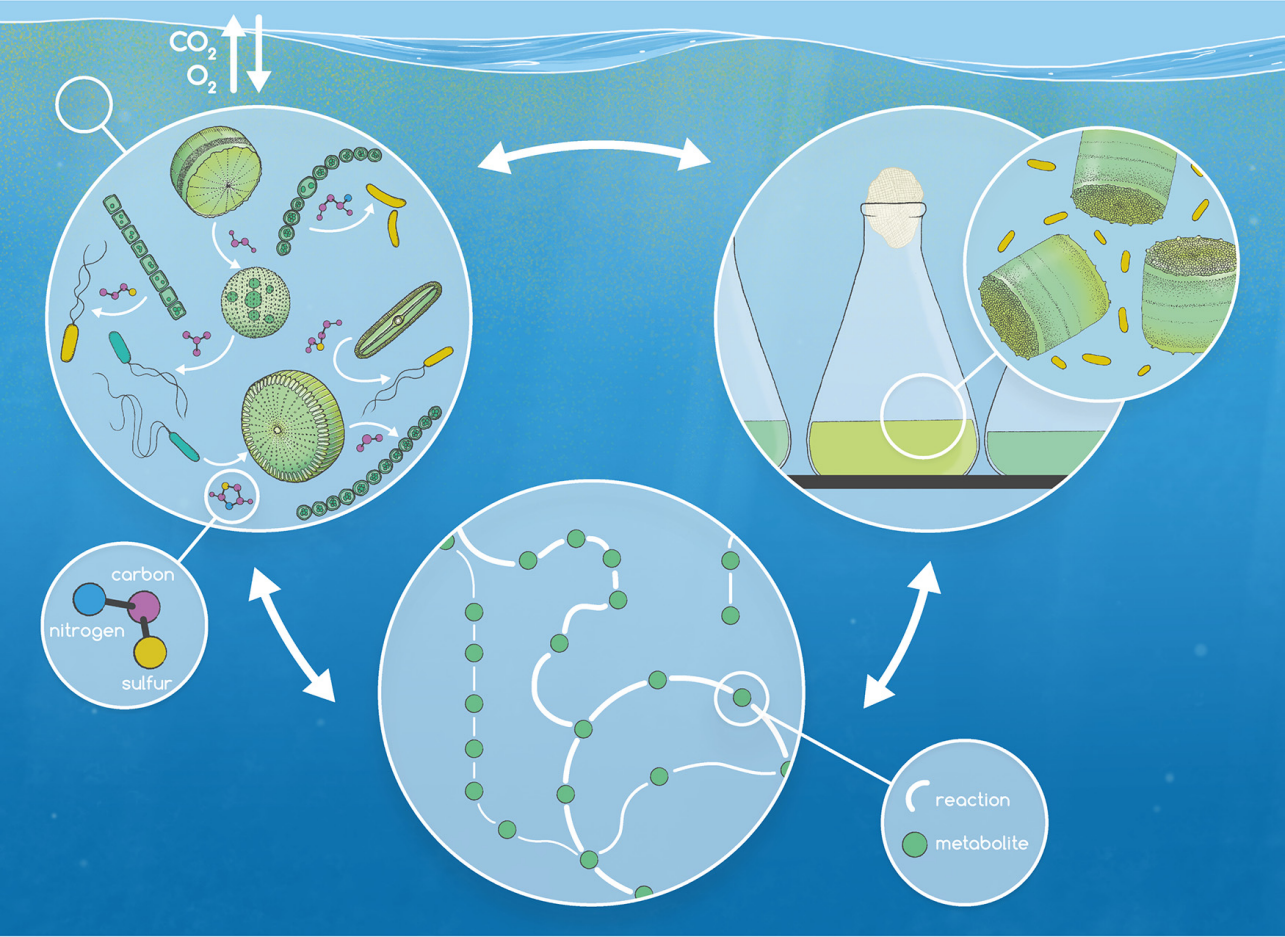
Microbial metabolic networks in the surface ocean are examined through complementary field-based observations (top left), laboratory coculture systems (top right), and metabolic modeling approaches (bottom center). (Top left) Phytoplankton and bacterial taxa form metabolic networks through the exchange of specific organic metabolites (purple) that include sulfur (yellow)- and/or nitrogen (blue)-containing molecules. Microbial transformation of organic matter ultimately controls ocean biogeochemistry as well as the balance of carbon storage in the ocean and carbon remineralization to CO_2_ through processes of photosynthesis and respiration. (Top right) To deconstruct microbial metabolic exchanges, coculture model systems have been useful for detecting taxon-specific metabolites that inform our interpretation of field observations. Likewise, field-based observations of community-level dynamics have generated hypotheses about metabolic exchange that can be tested with culture studies. (Bottom center) Metabolic modeling of microbial plankton is an emerging tool that can be integrated with culture and field studies to simulate and predict metabolic fluxes (denoted by thickness of lines) of metabolites (circles) during microbial interactions under specific conditions. While observational data are useful for constraining relevant parameters and improving model accuracy, modeling approaches aid in identifying gaps in our current knowledge that can be approached with future culture and field studies. Iterative application of these tools will improve our predictive capabilities for microbial activity under current and future ocean conditions. Figure illustration and design by Joana C. Carvalho (© Carvalho, printed with permission).

Marine ecosystems are dominated by microscopic organisms that make up over two-thirds of ocean biomass (1) and function as the major primary producers and consumers in the ocean. Each day, unicellular phytoplankton transform 100 million tons of inorganic carbon into organic substrates (2) that fuel the activity of heterotrophic bacteria (3), regulating the balance of carbon remineralization to CO_2_ and carbon storage in the ocean. Thousands of different molecules make up this microbially cycled pool of organic matter, forming the basis of intricate phytoplankton-bacterial interaction networks. However, linking individual metabolites to taxon-specific interactions is challenging due to the diversity of molecules and interacting organisms simultaneously present in seawater. My research group employs a combination of laboratory, computational, and field-based approaches to decipher currencies and regulatory mechanisms that establish phytoplankton-bacterial interactions (Fig. 1). Most notably, we have demonstrated the role of microbial sulfur-containing metabolites in influencing central biogeochemical reactions in the ocean (4–6). Overall, our pursuit in studying microbial metabolic exchange is centered on understanding how these micrometer-scale interactions impact ecosystem-scale community structure and the fate of elements and climate-active gases in the ocean and atmosphere.

Why costly metabolic interactions develop within microbial communities is a fundamental biological question. In examining metabolic exchange, we grapple with how microbial cooperation alters the rate of carbon-cycle-relevant reactions as well as ecosystem structure and stability. For instance, around half of eukaryotic phytoplankton are auxotrophic for vitamin B_12_ that they acquire from bacteria (7), and members of B_12_-requiring phytoplankton and B_12_-producing bacterial groups have evolutionary histories that point to complex metabolic interactions and coevolution (8). Indeed, coevolving microbes whose growth depends on reciprocal exchange of metabolites rapidly develop a costly metabolic cooperation where both partners increase metabolite production to benefit their corresponding partner (9). Increased metabolite production within interaction networks may also increase the robustness of these communities to ecological disturbance (10). Conversely, species do not have to evolve in a habitat to participate in interactions in that habitat. An organism exists wherever its realized fitness is great enough for persistence, through ecological fitting or readjustment to its habitat (11). Our observations likely reflect some combination of fitting and evolutionary processes that together have shaped microbial communities, with implications for ocean biogeochemistry and vulnerability to a changing climate.

## MODEL-SYSTEMS APPROACHES TO MICROBIAL INTERACTIONS

A central avenue of my research program is employing model microbial systems to study phytoplankton-bacterial interactions under controlled laboratory settings. Early in my career, I established a coculture system with the model diatom Thalassiosira pseudonana and model marine bacterium Ruegeria pomeroyi to act as representatives for phytoplankton-bacterial interactions in the ocean. I based the model system on vitamin B_12_ auxotrophy in the diatom, wherein the B_12_-producing bacterium provided the essential vitamin to the diatom in exchange for organic substrates as food. Using concurrent transcriptomic and metabolomic analyses, we identified sulfur- and nitrogen-containing metabolic currencies and signaling mechanisms that mediate their interaction (12–14). A number of newly recognized metabolites used in the marine microbial trade market are transformed through unidentified pathways likely regulated by changes in environmental landscape (e.g., light, nutrient availability, salinity, temperature). A clear step forward is to resolve unknown pathways and environmental regulators that influence the flux and timing of microbial metabolite exchange in our current and future ocean.

### Sulfur metabolites facilitate microbial cooperation.

Foremost, model-system studies have revealed sulfur-containing metabolites as important currencies in phytoplankton-bacterial interactions. I found that bacterial heterotrophy was supported by diatom-derived sulfonate molecules not previously recognized in marine systems (12). Additional model organism and metabolomics studies continue to reveal a chemical diversity of microbial sulfur metabolites (5, 15, 16). Concurrent with an expanding repertoire of newly recognized sulfur metabolites, the oceanography community has recognized a large inventory of dissolved organic sulfur in the ocean that is poorly characterized yet whose transformation influences ocean and atmospheric chemistry (4, 6). Sulfur-mediated interactions are a resourceful mechanism in marine ecosystems. Because sulfate concentration is high in seawater (∼28 mM), phytoplankton can leverage this readily available inorganic nutrient to shunt excess reducing power from photosynthesis, and bacterial associates use resulting organic sulfur outputs for growth. Indeed, phytoplankton-derived sulfur metabolites display day-night rhythms in abundance consistent with this idea (17, 18). Regulation of microbial sulfur metabolism is a current gap in our understanding of marine sulfur biogeochemistry that we are now poised to address. My group is currently examining the relationship between light availability, photochemistry, and sulfur metabolism in model diatoms and cocultures to quantify how photosynthetic outputs and redox state regulate production of sulfur metabolites and transfer to bacterial associates.

### Microbes participate in active partnerships.

A second important observation from model systems has been recognition and signaling between partners. I identified a bacterial recognition system in *T. pseudonana* akin to that used in land plants to recognize bacteria (13). Bacteria also use plant hormones and antibiotics to alter phytoplankton cell cycle and growth rate (15, 19). Together, these findings have transformed the historical view of passive interaction among microbes through indirect excretion of metabolites (20) to one in which phytoplankton and bacteria actively adjust their physiology and metabolism to establish partnerships (16). We are currently working with metabolomes from a large culture data set to taxonomically resolve putative signaling molecules in marine microbes, with the idea that these metabolites can be assayed to determine their specific signaling functions.

## BRIDGING MODEL SYSTEMS AND FIELD OBSERVATIONS

Our group also aims to bridge findings from “simple” lab systems with observations of “complex” natural systems, using each to inform the other in an iterative process. The *T. pseudonana-R. pomeroyi* interaction provided a scaffold on which we characterized a suite of structurally related sulfonates in >30 marine microbial taxa. We then bioinformatically inferred metabolic pathways and tracked sulfonate dynamics in natural microbial communities of the North Pacific (17). So far, field observations suggest sulfonate production in phytoplankton is regulated by both light and nutrient availability (17, 18, 21), ideas that we are exploring back in the lab.

In a more expansive analysis of >300 metabolites from microbial culture and field data, we showed that phytoplankton taxonomy imparts a strong influence on the chemical composition of ocean metabolomes (21) with many of these metabolites likely to serve as the substrates in phytoplankton-bacterial exchange. Indeed, bacterial community assembly can be predicted by use of individual phytoplankton-derived metabolites (22, 23). One might say that phytoplankton “farm” bacteria through excreted metabolites, a mechanism that favors evolution of cooperative interactions. Currently, our group is using environmental metatranscriptomics and metabolomics data to identify links between community composition and metabolite exchange across environmental nutrient gradients. Notably, over half of the >300 metabolites detected by Heal et al. (21) could not be identified. It is likely that additional carbon-cycle-relevant currencies are within these unidentified molecules, and continued effort to identify these metabolites is critical. For as long as these molecules remain unrecognized microbial currencies, particularly when abundant plankton groups are involved, they represent gaps in our reconstruction of marine elemental cycles.

## FUTURE OUTLOOK: METABOLIC MODELING APPROACHES AND PARALLELS TO OTHER BIOLOGICAL SYSTEMS

We cannot test a model organism system under all conditions and their intersections relevant to the natural environment. Our observations represent snapshots of interacting partners in time under a particular set of conditions. On the other hand, with field measurements we face the challenge of disentangling multiple, interacting environmental conditions that affect microbial partnerships. With this realization, I have been inspired by the work of van Tol and Armbrust (24) and others in building metabolic models of marine plankton. Metabolic modeling is a fresh lens through which to study marine microbial metabolism, allowing us to test microbes under a more exhaustive and interacting set of conditions that will inform future experiments. Metabolic modeling of *T. pseudonana* is already pointing us to gaps in our knowledge of respiration and sulfur and nitrogen metabolism that we need to constrain to improve quantitative predictions of metabolite exchange (24). In particular, the work of van Tol and Armbrust (24) shows a coupling of nitrogen and sulfur metabolism in balancing redox state in diatoms, which subsequently impacts the chemistry of organic substrates available to bacterial associates. Looking forward, our team plans to integrate culture studies, field observations, and metabolic models to more accurately quantify microbial metabolic exchange (Fig. 1).

Microbial cooperation is ubiquitous across ecosystems and has played pivotal roles in shaping the life and biogeochemistry of Earth. A reoccurring theme over the course of my research path has been observation of parallels between marine and terrestrial interactions. For instance, use of plant hormones in the mutualistic diatom-bacterial interactions (15) and plant-like bacterial recognition in the *T. pseudonana-R. pomeroyi* interaction (13) point to signaling analogs. Further, sulfonates have been implicated in terrestrial cyanobacterium-moss symbioses (25). Our group is beginning to extend studies into terrestrial microbial model systems, and we are thrilled about the potential overlaps and contrasts we might find across ecosystems. There are clear cross-ecosystem currencies at play, and identifying how currencies operate in different ecosystems may help us to establish the evolutionary mechanisms and ecological “rules” of cooperative metabolic trade.
